# Effectiveness and safety of opicapone in Parkinson’s disease patients with motor fluctuations: the OPTIPARK open-label study

**DOI:** 10.1186/s40035-020-00187-1

**Published:** 2020-03-04

**Authors:** Heinz Reichmann, Andrew Lees, José-Francisco Rocha, Diogo Magalhães, Patrício Soares-da-Silva, the OPTIPARK investigators, Csaba Antal Zolnai, Claudius Bartels, Andreas Barth, Kriemhild Barth, Stephan Behrens, Arnfin Bergmann, Ralf Bodenschatz, Rommy Born, Moriz Brandt, Sebastian Brock, Bernd Brockmeier, Christof Brücke, Norbert Brüggemann, Bernhard Bühler, Uwe Bungard, Lukas Cepek, Ilona Csoti, Max Deist, Carl Detlev Reimers, Ulrich Dölle, Sylke Domke, Imanuel Dzialowski, Georg Ebersbach, Heike Eggert, Karla Eggert, Reinhard Ehret, Jana Engel, Urban Fietzek, Anke Friedrich, Michael Fritzinger, Florin Gandor, Klaus Gehring, Stephan Gierer, Stephanie Gierer, Vasil Gjaurov, Doreen Gruber, Özkan Günes, Thomas Haas, Kirsten Hahn, Anna Eszter Haraszti, Rolf Hartmann, Bernhard Haslinger, Eva Heiss, Heinz P. Herbst, Frank Hoffmann, Werner E. Hofmann, Günter Höglinger, Wolfgang Jost, Anna-Maria Kavcic, Christoph Kellinghaus, Bertold Klemperer, Fabian Klostermann, Thomas Knoll, Natalia Koleva-Alazeh, Jiri Koschel, Diana Waltraud Kraft-Safavi, Almut Kronenberger, Andrea Kühn, Andreas Kupsch, Thomas Lehnhoff, Peter Laumen, Paul Lingor, Karla Lippmann, Michael Lorrain, Fabian Maass, Siegfried Muhlack, Thomas Müller, Michael Nagel, Stephan Neudecker, Katja Odin, Christian Oehlwein, Hakan Orbasli, Wolfram von Pannwitz, Heidi Pape, Robert Pfister, Tino Prell, Reinhard Puzich, Daniela Rau, Rene Reese, Gerd Reifschneider, Gernot Reimann, Stefani Ries, Christoph Rieth, Charlotte Rewitzer, Ali Safavi, Alexander B. Schmied, Johannes Schwarz, Wolfgang Schwarz, Joachim Springub, Inga Suttrup Claus, Vera Tadic, Klaus Tiel-Wilck, Lars Tönges, Jens Tröger, Christoph Schrey, Alexander Schulze, Sven Thonke, Tobias Wächter, Achim S. Wannenmacher, Tobias Warnecke, Bettina Wieder, Martin Wimmer, Christian Winkler, Otto Witte, Dirk Woitalla, Samis Zella, Uwe Ziebold, Jane Alty, Reem Amin, Michaela Boca, Stephen Butterworth, Camille Carroll, Gavin Charlesworth, K. Ray Chaudhuri, Rajkumar Chinnadurai, Jemima Collins, Jeremy Stephen Cosgrove, Samantha Cravey, Dinesh Damodaran, Nikolay Dimitrov, Rory Durcan, Simon Ellis, Adbdul Elmarimi, Jonathan Evans, James Fisher, Donald Grosset, Stuart Jamieson, Christopher Kobylecki, Sze Hway Lim, Veronica Lyell, Biju Mohamed, Sophie Molloy, Nicola Pavese, Dominic Paviour, Madeleine Purchas, Khalid Rashed, Christopher Rickards, Tabish Saifee, Gillian Sare, Christine Schofield, Naveen Setty, Jagdish Sharma, Ray Sheridan, Siew Lee Shu, Monty Silverdale, Rani Sophia, Sarah Statton, Malcolm Steiger, Christopher Thomas, Richard Walker, Tai Yen Foung

**Affiliations:** 1 Department of Neurology, University of Dresden, Dresden, Germany; 2 University College London, Reta Lila Weston Institute, London, UK; 3 Global Parkinson’s Disease Department, BIAL – Portela & CA S.A, Coronado, Portugal; 4 Research and Development Department, BIAL – Portela & CA S.A, da Siderurgia Nacional, 4745-457 S Mamede do Coronado, Portugal; 5 Department of Pharmacology and Therapeutics, Faculty of Medicine, University Porto, Porto, Portugal; 6 MedInUP, Center for Drug Discovery and Innovative Medicines, University Porto, Porto, Portugal

**Keywords:** Levodopa, Motor fluctuations, Open-label, Opicapone, Parkinson’s disease

## Abstract

**Background:**

The efficacy and safety of opicapone, a once-daily catechol-O-methyltransferase inhibitor, have been established in two large randomized, placebo-controlled, multinational pivotal trials. Still, clinical evidence from routine practice is needed to complement the data from the pivotal trials.

**Methods:**

OPTIPARK (NCT02847442) was a prospective, open-label, single-arm trial conducted in Germany and the UK under clinical practice conditions. Patients with Parkinson’s disease and motor fluctuations were treated with opicapone 50 mg for 3 (Germany) or 6 (UK) months in addition to their current levodopa and other antiparkinsonian treatments. The primary endpoint was the Clinician’s Global Impression of Change (CGI-C) after 3 months. Secondary assessments included Patient Global Impressions of Change (PGI-C), the Unified Parkinson’s Disease Rating Scale (UPDRS), Parkinson’s Disease Questionnaire (PDQ-8), and the Non-Motor Symptoms Scale (NMSS). Safety assessments included evaluation of treatment-emergent adverse events (TEAEs) and serious adverse events (SAEs).

**Results:**

Of the 506 patients enrolled, 495 (97.8%) took at least one dose of opicapone. Of these, 393 (79.4%) patients completed 3 months of treatment. Overall, 71.3 and 76.9% of patients experienced any improvement on CGI-C and PGI-C after 3 months, respectively (full analysis set). At 6 months, for UK subgroup only (*n* = 95), 85.3% of patients were judged by investigators as improved since commencing treatment. UPDRS scores at 3 months showed statistically significant improvements in activities of daily living during OFF (mean ± SD change from baseline: − 3.0 ± 4.6, *p* < 0.0001) and motor scores during ON (− 4.6 ± 8.1, *p* < 0.0001). The mean ± SD improvements of − 3.4 ± 12.8 points for PDQ-8 and -6.8 ± 19.7 points for NMSS were statistically significant versus baseline (both *p* < 0.0001). Most of TEAEs (94.8% of events) were of mild or moderate intensity. TEAEs considered to be at least possibly related to opicapone were reported for 45.1% of patients, with dyskinesia (11.5%) and dry mouth (6.5%) being the most frequently reported. Serious TEAEs considered at least possibly related to opicapone were reported for 1.4% of patients.

**Conclusions:**

Opicapone 50 mg was effective and generally well-tolerated in PD patients with motor fluctuations treated in clinical practice.

**Trial registration:**

Registered in July 2016 at clinicaltrials.gov (NCT02847442).

## Introduction

The success of levodopa used together with other antiparkinsonian drug classes means that most patients living with Parkinson’s disease (PD) enjoy a good quality of life for many years [1, 2]. Nevertheless, the long term therapeutic response is marred in many by the emergence of disabling fluctuations and dyskinesias [3, 4] that lead to a reduced quality of life and motor handicap [2, 5]. Wearing-off results from levodopa’s short duration response which reflects the amino acid’s short half-life (~ 60–90 min) [6]. Over time, patients will experience more and more hours per day in a disabling OFF-state and some will develop intrusive and adventitious involuntary movements [7].

Current treatment guidelines consider adjunctive treatment with catechol-*O-*methyltransferase (COMT) inhibitors, dopamine agonists and monoamine oxidase type B (MAO-B) inhibitors, as efficacious to reduce OFF time in patients treated with levodopa/dopa decarboxylase inhibitor (DDCI) therapy [8–10]. In routine practice, many physicians will also consider various formulations of levodopa (e.g. controlled-release and extended-release preparations) as well as dosing manipulations to increase the dose and/or dosing frequency of levodopa. COMT inhibitors have been an established first-line strategy to manage motor fluctuations for over 25 years [11–14], and are the only adjunct class to directly address the peak-trough variations in plasma levodopa levels that clinically manifest as wearing-off fluctuations [15]. The third generation COMT inhibitor – opicapone (Ongentys®, BIAL-Portela & Cª, S.A. Portugal) – has been approved in Europe since 2016 as adjunct therapy to preparations of levodopa/DDCI for end-of-dose motor fluctuations. Based on rational drug design, opicapone was specifically developed to reduce the risk of toxicity and improve peripheral tissue selectivity [16]. In one pharmacokinetic study, opicapone (50 mg once daily) significantly increased levodopa bioavailability compared with both placebo and entacapone (200 mg TID) by increasing substantially the trough plasma levels and each dose systemic exposure time (half-life) by at least 1 h [17]. Phase III studies have established that treatment with opicapone 50 mg once daily reduces daily OFF-time, without significantly increasing ON-time with troublesome dyskinesia versus placebo, and most patients show an improvement in the Clinician’s Global Impression of Change (CGI-C) [18, 19].

While placebo-controlled trials remain the gold standard in assessing response to a therapeutic intervention, alone they do not provide sufficient information of clinical effectiveness and safety. Many regulators and payers now encourage the supplementation of randomized controlled trials with other forms of evidence, such as ‘real-world’ studies [20, 21]. The aim of this study was to evaluate the change in the patient’s perception about his/her global PD condition (as assessed by CGI-C) after 3 months of routine treatment in clinical practice with once-daily opicapone 50 mg.

## Methods

### Study conduct

This was a prospective open-label, single-arm, multicenter trial evaluating opicapone 50 mg effectiveness in levodopa-treated PD patients experiencing motor fluctuations. The study was conducted from November 2016 to July 2018 at 68 specialist neurology centers in Germany and the United Kingdom (UK). Institutional review boards at the participating sites approved the protocol and the trial was conducted in accordance with the Declaration of Helsinki and International Conference on Harmonization Good Clinical Practice Guidelines. All patients provided written informed consent; the study was registered at EudraCT (2016–002391-27) and clinicaltrials.gov (NCT02847442).

### Study population

Men and women (≥30 years) with idiopathic PD [22] were eligible if they reported symptoms of motor fluctuations as identified by at least one symptom on the 9-Symptom Wearing-off Questionnaire (WOQ-9) [23]. They also had to be Hoehn and Yahr stages I-IV (during ON) and treated with 3–7 daily doses of levodopa/DDCI. Key exclusion criteria were atypical parkinsonism, severe unpredictable OFF periods (investigator judgment) and severe hepatic impairment (Child-Pugh Class C). Patients previously or currently treated with tolcapone and/or opicapone were also excluded from the study, as were patients treated with MAO-A and MAO-B inhibitors other than those for the treatment of PD [i.e. selegiline (≤10 mg/day in oral formulation or ≤ 1.25 mg/day in buccal formulation), rasagiline (≤1 mg/day) or safinamide (≤100 mg/day)]. Patients treated with levodopa/DDCI/entacapone before trial entry were to discontinue entacapone at the baseline visit and switch to a levodopa/DDCI formulation. Likewise, separate entacapone was to be discontinued by the baseline visit at the latest.

### Study design

Patients received opicapone 50 mg capsules once-daily at bedtime, at least 1 hour before or after the last daily dose of levodopa/DDCI. The total duration of treatment was 6 months in UK sites and 3 months in German sites. The longer duration of study in the UK was to provide data for economic modelling which will be reported elsewhere. Investigators were free to adjust total daily levodopa/DDCI doses according to individual need after the baseline visit. At study end, patients could be prescribed with further opicapone treatment according to local standard practice.

Endpoints were assessed at baseline, 1 month and 3 months or at any early discontinuation visit, and patients in the UK were also assessed at 6 months. Best efforts were made to have the same investigator/rater per patient throughout the study. The primary outcome was the CGI-C (7 point scale, from very much improved to very much worse), which assessed the patient’s perception about his/her global PD condition after 3 months of treatment with opicapone 50 mg; the same rater assessed CGI-C throughout the study and before the patient made his/her own assessment. Secondary assessments included the Patient’s Global Impression of Change (PGI-C), WOQ-9 assessments, the Unified Parkinson’s Disease Rating Scale (UPDRS) sections I-IV [24], the Parkinson’s Disease Questionnaire (PDQ-8) [25], the Non-motor Symptoms Scale (NMSS) [26] and change from baseline in total daily levodopa dose and dosing frequency. Treatment compliance was calculated based on the numbers of dispensed and returned opicapone capsules and treatment duration excluding interruptions to study medication.

Safety was assessed through reporting of treatment emergent adverse events (TEAEs) as well as vital signs and physical and neurological examinations. Prespecified subgroup analyses also evaluated change from baseline in levodopa total daily dose in patients who reported dopaminergic adverse events (i.e. dyskinesia, nausea, vomiting, orthostatic hypotension, any hallucination, illusion, delusion or disturbance in attention).

### Statistical analysis

No sample size estimation was performed for this open-label study. The safety population included all patients who received ≥1 dose of opicapone. Effectiveness was assessed in the full analysis set which included all patients in the safety population who had ≥1 CGI-C recorded post-baseline. Analyses were primarily descriptive; missing values for the primary outcome measure (CGI-C) at Visit 4 was imputed using the last observation carried forward method. For UPDRS II (at ON and OFF), UPDRS III (at ON), UPDRS II plus III (at ON), PDQ-8 and NMSS (including each domain) the means of changes from baseline were analyzed using Student’s t-test.

In addition, subgroup analyses for the primary endpoint were performed by age (above vs. below the baseline mean age), baseline use of dopamine agonists and dopamine agonists plus MAO-B inhibitors (yes/no).

## Results

### Patient disposition and baseline characteristics

**Fig. 1:**
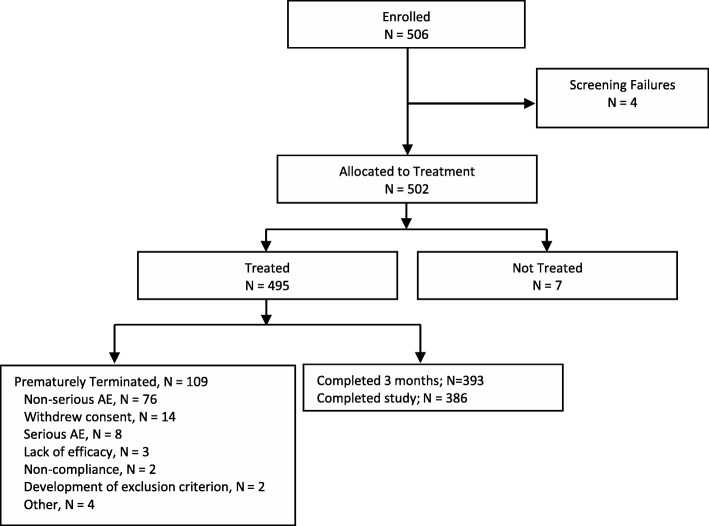
Patient disposition

Five-hundred and six patients were enrolled at 68 centers across Germany and the UK. Of these, 495 (97.8%) took at least one dose of opicapone (safety set) and 477 (94.3%) patients had at least one post-baseline CGI-C assessment and were included in the full analysis set (Fig. 1). A total of 109 (21.5%) patients prematurely terminated the trial and stopped treatment with opicapone; 84 patients (17.0%) withdrew due to a TEAE (including 13.3% [*n* = 66] due to an at least possibly related TEAE) while three (0.6%) withdrew because of lack of efficacy. A high percentage of patients (457/495; 92.3%) complied with ≥80% of doses. The mean ± SD treatment compliance was 99.2 ± 8.14%. Of the 386 patients who completed the trial, 332 patients (69.9%) continued to receive opicapone by prescription.

Baseline characteristics are provided in Table 1. The study population was comprised of white Caucasian patients with a mean ± SD age of 67.7 ± 8.98 years, a mean ± SD time since diagnosis of 102.1 ± 59.6 months and a mean ± SD duration of motor fluctuations of 30.1 ± 38.0 months. The majority of patients (78.8%) were on another levodopa adjunct medication: the most common reported adjunct medications were rasagiline (27.5%), pramipexole (24.8%) and ropinirole (22.4%).

**Table 1:** Baseline characteristics (safety set)

Category	*N* = 495
Age (years); mean ± SD [range]	67.7 ± 8.98 [43–87]
Age categories; n (%)	
≥30 to < 65	164 (33.1)
≥65 to < 85	325 (65.7)
≥85	6 (1.2)
Sex (M/F); n (%)^a^	315 (63.6)/179 (36.2)
Race: n (%)	
White	495 (100.0)
Duration of Parkinson’s disease (months);	
Mean ± SD	102.1 ± 59.60
Median [range]	89 [5–420]
Duration of motor fluctuations (months);	
Mean ± SD	30.1 ± 37.97
Median [range]	15 [0–324]
Symptoms (WOQ-9 assessment); n (%)^b^	
Tremor	299 (62.7)
Any slowness of movement	459 (96.2)
Mood changes	248 (52.0)
Any stiffness	393 (82.4)
Pain/aching	286 (60.0)
Reduced dexterity	433 (90.8)
Cloudy mind/slowness of thinking	223 (46.8)
Anxiety/panic attacks	119 (24.9)
Muscle cramping	288 (60.4)
Total levodopa daily dose (mg); mean ± SD	580.1 ± 289.1
Median [range]	525.0 [100–3750]
Adjunct therapies; n (%)^c^	
Rasagiline	136 (27.5)
Pramipexole	123 (24.8)
Ropinirole	111 (22.4)
Amantadine	105 (21.2)
Rotigotine	68 (13.7)
Safinamide	67 (13.5)
Piribedil	44 (8.9)

^a^*n* = 1 missing, ^b^assessed in the full analysis set, ^c^patients could take ≥ 1 adjunct therapy

### Clinician and patient global impressions of change

**Fig. 2:**
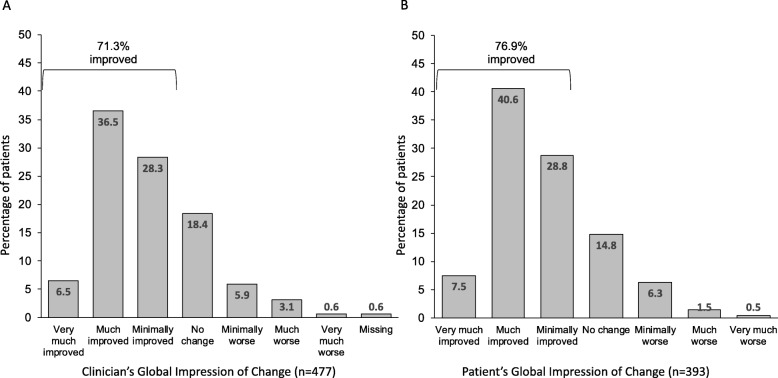
Global Impression of Change following 3 months treatment with opicapone 50 mg (LOCF) (**a**) investigator rated (CGI-C, *n* = 477); (**b**) self-rated by the patient (PGI-C, *n* = 393)

After 3 months of treatment with opicapone 50 mg, the majority of patients (71.3%) showed clinical improvement as judged by the investigators (CGI-C), with 43% reported as much or very much improved (Fig. 2a).

Similar improvements had already been observed at the earlier 1 month timepoint, where 75.8% of patients were judged as improved, 16.9% as having ‘no change’ and 6.6% as having worsened. For those UK patients (*n* = 95) who were also assessed at 6 months, 85.3% were judged as improved since commencing treatment (8.5% very much improved and 49.4% much improved) while 8.5% were judged as showing ‘no change’ and 6.6% as having worsened. Patients self-rated levels of improvement (PGI-C) were consistent with primary results, with the majority of patients (76.9%) reporting an improvement after 3 months of treatment with opicapone 50 mg (Fig. 2b). For the subgroup of UK patients also assessed at 6 months (*n* = 94), 79.8% self-reported an improvement, 12.8% reported no change and 7.5% reported worsening.

Subgroup analyses for the primary efficacy endpoint (CGI-C at Month 3) confirmed that improvements were generally seen regardless of age or concomitant use of dopamine agonists with or without MAO-B inhibitors at baseline (Supplementary Table 1, Additional file 1).

### Presence of symptoms as assessed by the WOQ-9

**Fig. 3:**
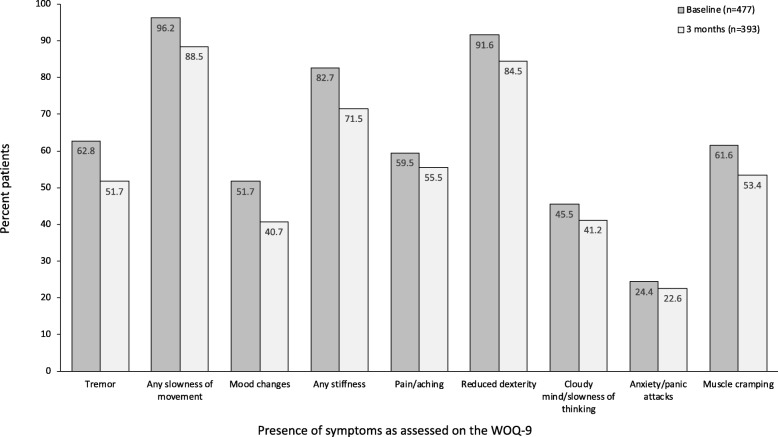
Presence of PD symptoms as assessed on the WOQ-9 in patients who completed 3 months of study

The proportions of patients reporting the overall presence of individual symptoms on the WOQ-9 decreased from baseline to 3 months (Fig. 3).

### Rating scale outcomes

Assessments of UPDRS scores after 3 months of opicapone treatment showed stability of mentation, behavior and mood (Part I scores) and statistically significant improvements in activities of daily living (ADL, Part II) during OFF, motor scores (Part III) during ON and Total scores (Parts II + III) during ON (Table 2). After 3 months of treatment, there was a mean reduction of 0.8 ± 1.9 points in UPDRS IV scores (complications of therapy in the past week).

**Table 2:** Rating scale assessments

Rating scale	
UPDRS Part I (mentation, behavior and mood); mean ± SD	
Baseline (*n* = 477)	2.3 ± 2.1
3 months (*n* = 393)	1.9 ± 1.9
Change from baseline (*n* = 393)	− 0.3 ± 1.5
UPDRS Part II (ADL during OFF); mean ± SD	
Baseline (*n* = 476)	17.1 ± 7.0
3 months (*n* = 391)	13.9 ± 6.8
Change from baseline (*n* = 391)	− 3.0 ± 4.6
*P* value vs. baseline	< 0.0001
UPDRS Part III (motor scores during ON); mean ± SD	
Baseline (*n* = 477)	26.5 ± 12.1
3 months (*n* = 393)	21.5 ± 11.0
Change from baseline (*n* = 393)	− 4.6 ± 8.1
*P* value vs. baseline	< 0.0001
UPDRS Total scores (Part II + III); mean ± SD	
Baseline (*n* = 477)	37.5 ± 16.9
3 months (*n* = 393)	30.5 ± 15.2
Change from baseline (*n* = 393)	− 6.4 ± 10.4
*P* value vs. baseline	< 0.0001
UPDRS Part IV (complications of therapy); mean ± SD	
Baseline (*n* = 475)	5.2 ± 2.6
3 months (*n* = 391)	4.2 ± 2.4
Change from baseline (*n* = 391)	− 0.8 ± 1.9
PDQ-8 Total Score; mean ± SD	
Baseline (*n* = 476)	29.2 ± 16.4
3 months (*n* = 393)	25.2 ± 15.8
Change from baseline (*n* = 393)	− 3.4 ± 12.8
*P* value vs. baseline	< 0.0001
NMSS Score; mean ± SD	
Baseline (*n* = 477)	44.6 ± 30.3
3 months (*n* = 393)	37.0 ± 26.7
Change from baseline (*n* = 393)	− 6.8 ± 19.7
*P* value vs. baseline	< 0.0001

*NMSS* Non-motor symptom scale, *UPDRS* Unified Parkinson’s Disease Rating Scale, *PDQ-8* Parkinson’s Disease Questionnaire

Improvements in both patient quality of life (as assessed by the PDQ-8) and non-motor symptoms (as assessed by the NMSS) were also observed after 3 months of treatment with opicapone. The mean ± SD improvements of − 3.4 ± 12.8 points for PDQ-8 and -6.8 ± 19.7 points for NMSS were statistically significant versus baseline (both *p* < 0.0001). For the NMSS, most domains either remained stable or showed improvement versus baseline (Supplementary Table 2, Additional file 1). For example, sleep/fatigue showed a mean ± SD change from baseline of − 1.3 ± 6.3 points (*p* < 0.0001) and mood/cognition showed a mean ± SD change from baseline of − 1.5 ± 6.82 points; *p* < 0.0001).

### Levodopa dosing

After 3 months of treatment with opicapone, most patients remained on the same total daily levodopa dose (85% had no change in dose, 8.6% had a dose increase and 5.7% had a dose decrease) and levodopa dosing frequency (77.1% had no change, 8.4% had an increase and 12.0% had a decrease in dosing frequency [data missing for 2.4%]), resulting in an overall mean change of approximately − 10 mg/day. Similarly, for patients who reported dopaminergic adverse events (full analysis set), most patients (62.7%) remained on the same total daily levodopa dose, 14.5% had a dose increase and 22.7% had a dose decrease, resulting in an overall mean change of − 26.6 mg/day.

### Safety and tolerability

Overall, 371 (74.9%) patients experienced TEAEs, which were mostly assessed as mild or moderate (Table 3). Thirty four (6.9%) patients experienced serious TEAEs, including one death due to endocarditis that was considered unrelated to treatment. A total of 223 (45.1%) patients reported TEAEs that were assessed as at least possibly related to treatment. In line with the pivotal studies, the most frequent TEAEs (> 5%) considered possibly treatment-related were dyskinesia (11.5%), dry mouth (6.5%) and dizziness (4.8%); diarrhea was reported in 3 (0,6%) patients. The frequency of at least possibly related serious TEAEs was low: seven patients (1.4%) had ≥1 of these events - anxiety, visual hallucination, psychotic disorder, dizziness, hypertension, hypotension, tachycardia and femoral neck fracture.

**Table 3:** Incidence of treatment emergent adverse events

TEAE Category	*N* = 495
Any TEAE	371 (74.9)
Any treatment-related^a^ TEAE	223 (45.1)
Any serious TEAE	34 (6.9)
Any treatment-related^a^ serious TEAE	7 (1.4)
Any TEAE leading to discontinuation	84 (17.0)
Any treatment-related^a^ TEAE leading to discontinuation	66 (13.3)
Any serious TEAE leading to discontinuation	8 (1.6)
Any TEAE leading to death	1 (0.2)
Treatment-related TEAEs (≥2% patients)	
Dyskinesia	57 (11.5)
Dry mouth	32 (6.5)
Dizziness	24 (4.8)
Nausea	22 (4.4)
Constipation	20 (4.0)
Insomnia	12 (2.4)
Hallucination	11 (2.2)
Fall	10 (2.0)
TEAEs leading to discontinuation (≥1% patients)	
Nausea	10 (2.0)
Constipation	7 (1.4)
Hallucination	6 (1.2)
Dizziness	5 (1.0)
Dyskinesia	5 (1.0)

^a^Treatment-related TEAEs were any TEAEs that were considered at least possibly related by the investigator and include the events with missing relationship assessment

TEAEs led to premature termination in 84 (17.0%) patients, but the precipitating events were diverse: the most common TEAEs leading to withdrawal were nausea (2.0%) and constipation (1.4%). Of these, 66 patients (13.3%) had at least possibly treatment-related TEAEs leading to discontinuation. There were no relevant changes in vital signs, physical and neurological examinations throughout the study.

When analyzed by age (above vs. below the baseline mean age), of the 371 patients that experienced TEAEs, 56.6% were from the older group (≥67.7 years old). Patients older than 67.7 years old also accounted for the highest proportion of those who discontinued due to a TEAE (*n* = 57 of 84).

## Discussion

Taken overall, the results of this large open-label study in PD patients with motor fluctuations are the first to confirm the effectiveness, safety and tolerability of once daily opicapone 50 mg as used in routine clinical practice. The majority of patients showed improvements in their perception about global PD condition (≥70% as judged by clinicians and the patients themselves) 3 months after they started treatment with opicapone 50 mg. Treatment was generally well-tolerated and adverse events were as expected for a dopaminergic therapy in patients with PD.

While randomized controlled studies are often criticized as recruiting patients who might best meet study endpoints [27], this large study mirrored a clinical setting population by allowing the inclusion of a broader population of fluctuating PD patients, including patients with Hoehn and Yahr Stage IV. It is of interest to note that more patients were judged to have shown clinical improvement in this real life study than had been reported in the pivotal trials (71.3% in this study vs. 59.6% in the combined pivotal studies [28]). Even more notable was the proportion of patients who were judged as much/very much improved (43.0% in this study vs 25.2% in the combined pivotal studies [28]). These judgements made by the investigators were corroborated by the patients themselves with 40.5% patients reporting they were much or very much improved after 3 months treatment with opicapone 50 mg. Treatment with opicapone was also associated with a small but significant improvement in overall quality of life, as assessed using the PDQ-8. As also observed from the pivotal studies [18, 19], there was a proportion of patients who did not respond well to adjunct therapy with opicapone. Studies with entacapone have shown that the response to COMT inhibition is modulated by the COMT Val158Met polymorphism, with significantly enhanced efficacy in patients with the COMT (HH) genotype [29]. The impact of the COMT genotype on the opicapone response is unknown and merits further study.

Despite optimized anti-PD therapy (according to clinician’s judgement) and the fact that most (78.8%) patients were receiving levodopa plus another PD medication, UPDRS motor and ADL scores significantly improved (by 4.6 and 3.0 points, respectively) with opicapone as adjunct therapy. These magnitudes of effects have been reported to be clinically significant [30–32] and may therefore indicate that treatment with opicapone, not only increases ON time, but also improve the quality of ON time. Non-motor symptoms are now acknowledged as an important source of disability and contributor to worse quality of life for people living with PD [33, 34]. In line with prior pivotal studies with opicapone, this study also hinted towards an overall improvement in non-motor symptoms [18, 19]. Some non-motor domains are known to correlate with the motor OFF-state and be dopa-responsive [35, 36] – with the implication that optimization of the pharmacokinetic and pharmacodynamic profile of levodopa with opicapone may be beneficial in their management. As recently suggested by Fabbri et al. [37], the effect of opicapone on various non-motor symptoms merits further investigation.

Patients in this study maintained their levodopa dose for up to 6 months with sustained benefits in symptomatic control. Similar observations have also been seen in the 1-year-long extensions of the pivotal studies [28, 37, 38]. This hints at a possible long-term delay of need for levodopa increase.

Opicapone 50 mg was generally well-tolerated, with the majority of events reported as mild or moderate in severity. While the most common reason for withdrawal from the study was adverse events (17.0%), the causes were diverse, with the most frequent (nausea) affecting only 2% of patients (*n* = 10). While differences in study duration and settings make comparisons difficult, similar discontinuation rates due to AEs were reported in open-label studies with entacapone [39, 40]. Although dyskinesia was reported as a TEAE in 11.5% of patients, only five patients (1%) discontinued from the study due to dyskinesia. The rate of serious TEAEs considered at least possibly related to treatment was low (1.4%). One death was reported; the 69 year old male patient died due to severe endocarditis which was considered by the investigator not to be related to the study medication.

Strengths of this study lie in its size, broad inclusion criteria and routine practice setting. Although this study permitted inclusion of a broad range of disease severities (Hoehn and Yahr I-IV), we did not capture sufficient data in this pragmatic study to analyze by subgroups. Other weaknesses include those inherent to open-label studies without placebo control, where both the clinician and patient have expectations from treatment. Also, the study was only conducted in two countries (UK and Germany) and all patients were white Caucasian. We did not study OFF and ON time since patient diaries carry significant patient burden [41], which we wanted to minimize in this routine practice study.

## Conclusion

In routine clinical practice, once-daily opicapone 50 mg as adjunct to levodopa-treated PD patients with motor fluctuations significantly improved patients’ perception about their global PD condition. A similar impression was reported by the clinicians. Opicapone was generally well tolerated and significantly improved quality of life and both motor and non-motor symptoms. These findings confirm the clinical utility of opicapone 50 mg as an effective adjunct option for the management of motor fluctuations in levodopa-treated PD.

## Supplementary information

**Additional file 1: Table S1.** Subgroup analyses of the primary endpoint (CGI-C at Month 3) by age and concomitant use of other adjunct medications. **Table S2.** Change from baseline in NMSS domains.

## Data Availability

The study sponsor (BIAL) undertakes to share, upon request, anonymized patient-level, study-level clinical trial data (analyzable data sets), and other information (such as protocols) from this clinical trial to qualified researchers as necessary for conducting legitimate research. Information is provided at www.bial.com.

## Abbreviations

CGI-C: Clinician’s Global Impression of Change
COMT: catechol-*O-*methyltransferase
MAO-B: Monoamine oxidase type B
NMSS: Non-motor Symptoms Scale
PD: Parkinson’s disease
PDQ-8: Parkinson’s Disease Questionnaire
PGI-C: Patient’s Global Impression of Change
TEAEs: Treatment-emergent adverse events
UPDRS: Unified Parkinson’s Disease Rating Scale
WOQ-9: Wearing-off Questionnaire (nine items)
