# Correction to: Effectiveness and safety of opicapone in Parkinson’s disease patients with motor fluctuations: the OPTIPARK open-label study

**DOI:** 10.1186/s40035-020-00193-3

**Published:** 2020-04-28

**Authors:** Heinz Reichmann, Andrew Lees, José-Francisco Rocha, Diogo Magalhães, Patrício Soares-da-Silva, the OPTIPARK investigators

**Affiliations:** 1 Department of Neurology, University of Dresden, Dresden, Germany; 2 University College London, Reta Lila Weston Institute, London, UK; 3 Global Parkinson’s Disease Department, BIAL – Portela & CA S.A, Coronado, Portugal; 4 Research and Development Department, BIAL – Portela & CA S. A, da Siderurgia Nacional, 4745-457 S Mamede do Coronado, Portugal; 5 Department of Pharmacology and Therapeutics, Faculty of Medicine, University Porto, Porto, Portugal; 6 MedInUP, Center for Drug Discovery and Innovative Medicines, University Porto, Porto, Portugal

**Correction to: Transl Neurodegener (2020) 9:9**

**https://doi.org/10.1186/s40035-020-00187-1**

In this published article [1], the efficacy data for the below outcomes were unfortunately not supplied based on the Full Analysis Set (FAS) at 3 and/or 6 months for specific outcomes under the statistical analysis plan (SAP). The SAP specified that the efficacy data should be based overall on the FAS at 3 months for the primary (with last observation carried forward, LOCF) and secondary (no LOCF) efficacy endpoints and 6 months for UK only (no LOCF). The amendments do not change the conclusions of the paper, and the corrected data are given below.

**Clinician and patient global impressions of change**

***Correction to CGI-C 6-month data (UK patients only)***

[ … ] For those UK patients (*n* = 95) who were also assessed at 6 months, 85.3% were judged as improved since commencing treatment (8.4% very much improved and 49.5% much improved) while 8.4% were judged as showing ‘no change’ and 6.4% as having worsened. [ … ]

Correction to Fig. 2b

**Fig. 2:**
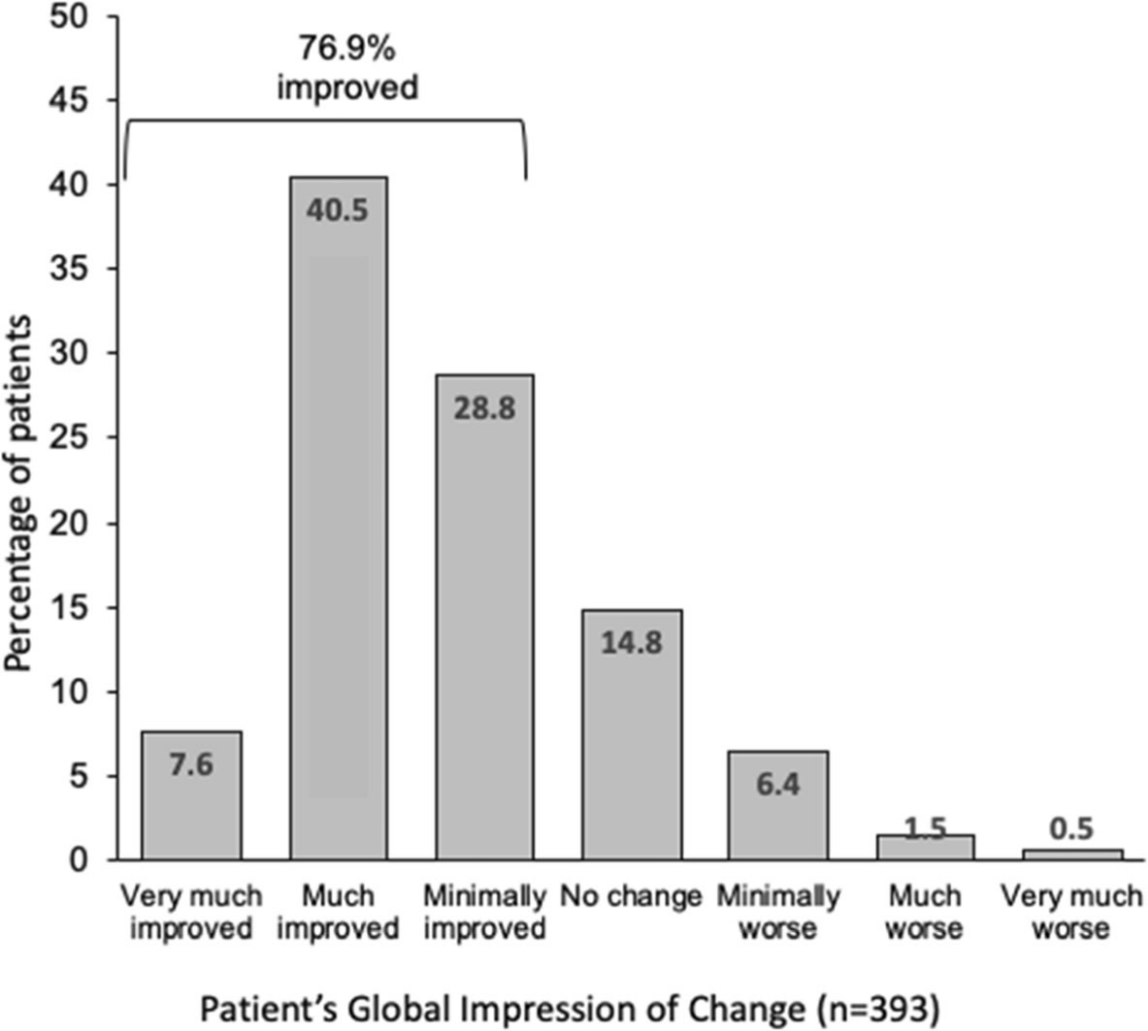
Global Impression of Change following 3 months treatment with opicapone 50 mg (a) investigator rated (CGI-C, *n* = 477, LOCF); (b) self-rated by the patient (PGI-C, *n* = 393)

**Rating scale outcomes**

***Correction to UPDRS analyses (Table*** ***2******)***

**Table 2:** Rating scale assessments

Rating scale	
[ … ]	
UPDRS Part II (ADL during OFF); mean ± SD	
Baseline (*n* = 475)	17.1 ± 7.0
3 months (*n* = 389)	13.9 ± 6.8
Change from baseline (*n* = 389)	− 3.0 ± 4.6
*P* value vs. baseline	< 0.0001
UPDRS Part III (motor scores during ON); mean ± SD	
Baseline (*n* = 476)	26.5 ± 12.1
3 months (*n* = 391)	21.5 ± 11.0
Change from baseline (*n* = 391)	− 4.6 ± 8.1
*P* value vs. baseline	< 0.0001
[…]	

*NMSS* Non-motor symptom scale, *UPDRS* Unified Parkinson’s Disease Rating Scale, *PDQ-8* Parkinson’s Disease Questionnaire

N numbers for UPDRS Part II were 475 at baseline and 389 at 3 months (Visit 4); results of the change from baseline analysis remain unchanged. N numbers for UPDRS Part III were 476 at baseline and 391 at 3 months; results of the change from baseline analyses remain unchanged.

***Correction to*** Table 2.

**Levodopa dosing**

***Correction to levodopa dosing***

After 3 months of treatment with opicapone, most patients remained on the same total daily levodopa frequency (77.1% had no change, 10.4% had an increase and 12.5% had a decrease in dosing frequency), resulting in an overall mean change of approximately − 10 mg/day. […].

**Discussion**

***Correction to discussion***

[ … ] These judgements made by the investigators were corroborated by the patients themselves.

with 48.1% patients reporting they were much or very much improved after 3 months treatment with opicapone 50 mg. [ … ]

**Supplementary material**

***Correction to*** Supplementary Table 2

Results of the change from baseline in perceptual problems/hallucinations was updated to 0.0 ± 2.01. Results from all other analyses remain unchanged.

Supplementary Table 2 Change from baseline in NMSS domains

**Table:** 

NMSS domain	Mean ± SD
[ … ]	
Perceptual problems/hallucinations	
Baseline	0.8 ± 2.45
Change from baseline (*p* value)	0.0 ± 2.01 (*p* = 0.7437)
[ … ]	
