# Supplementary material for: Effectiveness and safety of opicapone in Parkinson’s disease patients with motor fluctuations: the OPTIPARK open-label study
Source: Transl Neurodegener. 2020 Mar 4;9:9. doi: 10.1186/s40035-020-00187-1 (PMC7055125; doi:10.1186/s40035-020-00187-1)
Supplement: Supplementary file 1 — Additional file 1: Table S1. Subgroup analyses of the primary endpoint (CGI-C at Month 3) by age and concomitant use of other adjunct medications. Table S2. Change from baseline in NMSS domains. [file 40035_2020_187_MOESM1_ESM.docx]

**Additional File 1**

**Supplementary Table 1. Subgroup analyses of the primary endpoint (CGI-C at Month 3) by age and concomitant use of other adjunct medications at baseline**

| n (%) | Concomitant dopamine agonist use at baseline | | Concomitant dopamine agonist plus MAO-B use at baseline | | Age at baseline | |
| --- | --- | --- | --- | --- | --- | --- |
|  | Yes  N = 321 | No  N = 156 | Yes  N = 159 | No  N = 318 | <67.7 Years at Baseline  N = 209 | ≥67.7 Years at Baseline  N = 268 |
| Not assessed | 2 (0.6) | 1 (0.6) | 2 (1.3) | 1 (0.3) | 1 (0.5) | 2 (0.7) |
| Very much improved | 24 (7.5) | 7 (4.5) | 11 (6.9) | 20 (6.3) | 21 (10.0) | 10 (3.7) |
| Much improved | 125 (38.9) | 49 (31.4) | 68 (42.8) | 106 (33.3) | 83 (39.7) | 91 (34.0) |
| Minimally improved | 94 (29.3) | 41 (26.3) | 42 (26.4) | 93 (29.2) | 59 (28.2) | 76 (28.4) |
| No change | 49 (15.3) | 39 (25.0) | 22 (13.8) | 66 (20.8) | 30 (14.4) | 58 (21.6) |
| Minimally worse | 16 (5.0) | 12 (7.7) | 9 (5.7) | 19 (6.0) | 10 (4.8) | 18 (6.7) |
| Much worse | 10 (3.1) | 5 (3.2) | 4 (2.5) | 11 (3.5) | 3 (1.4) | 12 (4.5) |
| Very much worse | 1 (0.3) | 2 (1.3) | 1 (0.6) | 2 (0.6) | 2 (1.0) | 1 (0.4) |

**Supplementary Table 2. Change from baseline in NMSS domains**

| NMSS domain | Mean ± SD |
| --- | --- |
| Cardiovascular, including falls  Baseline  Change from baseline (p value) | 1.5 ± 2.38  -0.2 ± 2.07 (p=0.0310) |
| Sleep/fatigue  Baseline  Change from baseline (p value) | 9.0 ± 7.53  -1.3 ± 6.3 (p<0.0001) |
| Mood/cognition  Baseline  Change from baseline (p value) | 6.7 ± 9.80  -1.5 ± 6.82 (p<0.0001) |
| Perceptual problems/hallucinations  Baseline  Change from baseline (p value) | 0.8 ± 2.45  2.01 ± 17.0 (p=0.7437) |
| Attention/memory  Baseline  Change from baseline (p value) | 4.4 ± 5.43  -0.5 ± 3.95 (p=0.0209) |
| Gastrointestinal tract  Baseline  Change from baseline (p value) | 4.0 ± 4.68  -0.7 ± 3.62 (p=0.0002) |
| Urinary  Baseline  Change from baseline (p value) | 7.6 ± 7.68  -0.9 ± 5.89 (p=0.0033) |
| Sexual function  Baseline  Change from baseline (p value) | 2.5 ± 5.03  -0.4 ± 3.90 (p=0.0390) |
| Miscellaneous  Baseline  Change from baseline (p value) | 8.2 ± 7.30  -1.6 ± 6.01 (p<0.0001) |
